# Speckle reducing bilateral filter for cattle follicle segmentation

**DOI:** 10.1186/1471-2164-11-S2-S9

**Published:** 2010-11-02

**Authors:** Jinshan Tang, Shengwen Guo, Qingling Sun, Youping Deng, Dongfeng Zhou

**Affiliations:** 1 Image Processing and Bioimaging Research Laboratory, System Research Institute & Department of Advanced Technologies, Alcorn State University, Alcorn State, MS, USA; 2 School of Computing, University of Southern Mississippi, 118 College Drive, Hattiesburg, MS 39406-0001, USA; 3 SpecPro Inc, 3909 Halls Ferry Road, Vicksburg, MS 39180, USA; 4 The Fifth Hospital of Harbin, Harbin, Heilongjiang, China

## Abstract

**Background:**

Ultrasound imaging technology has wide applications in cattle reproduction and has been used to monitor individual follicles and determine the patterns of follicular development. However, the speckles in ultrasound images affect the post-processing, such as follicle segmentation and finally affect the measurement of the follicles. In order to reduce the effect of speckles, a bilateral filter is developed in this paper.

**Results:**

We develop a new bilateral filter for speckle reduction in ultrasound images for follicle segmentation and measurement. Different from the previous bilateral filters, the proposed bilateral filter uses normalized difference in the computation of the Gaussian intensity difference. We also present the results of follicle segmentation after speckle reduction. Experimental results on both synthetic images and real ultrasound images demonstrate the effectiveness of the proposed filter.

**Conclusions:**

Compared with the previous bilateral filters, the proposed bilateral filter can reduce speckles in both high-intensity regions and low intensity regions in ultrasound images. The segmentation of the follicles in the speckle reduced images by the proposed method has higher performance than the segmentation in the original ultrasound image, and the images filtered by Gaussian filter, the conventional bilateral filter respectively.

## Background

Ultrasound imaging technology has wide applications in cattle reproduction and has been used to monitor individual follicles and determine the patterns of follicular development [1-6]. The adoption of ultrasound imaging technology in cattle reproduction can provide an effective way to understand a number of issues on bovine reproductive cycle and its concurrent disorders [4]. For example, with the help of ultrasound imaging technology, it is now known that follicular growth occurs in wave-like patterns during each estrous cycle [7]. Ultrasound imaging technology also provides a tool for understanding the influence of dominant follicles on medium and small follicles [7].

In the applications of ultrasound imaging to monitoring individual follicles and determining the patterns of follicular development, the acquisition of the measurements of the individual follicles such as diameters, areas and perimeters is very important. In order to acquire the measurements of an individual follicle, image segmentation techniques are often used to extract the individual follicles. However, speckles in ultrasound images affect the segmentation and finally affect the measurement of the follicles. Speckle noise, seen as a granular structure, is caused by the interaction between the ultrasound waves and the scatters within the tissue [8]. The inherent nature of speckles makes its removal difficult. Speckle noise is not an additive noise, but is considered as a kind of multiplicative noise [9][10]. Many speckle reduction technologies have been proposed. In [11], a Laplacian pyramid-based nonlinear diffusion (LPND) is presented for medical ultrasound imaging. In the proposed method, the image is first decomposed into multi-layer Laplacian pyramid and speckles are removed by nonlinear diffusion filtering of bandpass ultrasound images in Laplacian pyramid domain. In [12], a nonlinear multiscale wavelet diffusion for speckle reduction is proposed. Speckles are suppressed by employing the iterative multiscale diffusion on the wavelet coefficients. In [9], a speckle reduction algorithm---speckle reducing anisotropic diffusion (SRAD) is proposed. The proposed algorithm has good performance in the preservation of edges and speckle reduction.

In this paper, we will investigate using bilateral filter to reduce the speckles in ultrasound images for cattle follicle segmentation. It is well known that bilateral filter has good performance in noise reduction and edge preservation. However, current existing bilateral filters are mainly used for additive noise reduction. It is not effective when it is applied to speckles, which are generally modelled as multiplicative noise. In order to solve this issue, we propose an adaptive bilateral filter, which can reduce the speckles effectively.

## Methods

### Bilateral filter

Bilateral filter was developed by Tomasi and Manduchi [13]. The basic idea of bilateral filter is to replace a pixel value in an image by a weighted mean of its neighbors, which the weights depend on both the spatial distance and the intensity distance [14][15]. There are many types of bilateral filters depending on the choice of weighting functions. What we develop in this paper is based on the Gaussian bilateral filter [13][16]. For Gaussian bilateral filter, it can be expressed mathematically as [13][17]

     (1)

where  is the output pixel value, *J* (*Y*) is the input pixel values, *X* and *Y* are the coordinates vectors,* σ _d_*^2^ and *σ _r_*^2^ are the parameters controlling the fall-off of weights in spatial and intensity domains, respectively, *N*(*X*) is a spatial neighborhood of pixel *J*(*X*), || || is Euclidean distance, C is used for the normalization and is expressed as [13][17]

     (2)

In the above equation, when *X* and *Y* are 2-D vectors, the bilateral filter is called 2-D bilateral filter, which can be used to reduce the noise in 2-D images.

Bilateral filter is a good choice for image de-noising because it is stable and simple. The effectiveness of bilateral filter lies in the combined use of the domain filter, which is used to enforce spatial closeness by weighting pixel values with coefficients that fall off with distance [18], and the range filter, which assigns greater coefficients to those neighbouring pixels with light intensity that is more similar to the centre pixel value [18]. In bilateral filter, the choice of the parameters *σ _d_*^2^ and *σ _r_*^2^ is very important. If their values are set too high, the filter will act as a smoothing filter and will blur the edges. If their values are set too low, the noise cannot be removed. Generally speaking, the choice of *σ _d_*^2^ and *σ _r_*^2^ depends on the variance of the noise. Based on the research in [17], the optimal *σ _d_*^2^ is relatively insensitive to noise variance while the optimal *σ _r_*^2^ changes significantly as the noise standard deviation changes [17]. [17] also demonstrates that *σ _r_*^2^ and noise variance are linearly related to a large degree. The research in [17] is based on additive noise model. If bilateral filter is applied to speckle noise, the relationship between *σ _r_*^2^ and noise variance will not be established because speckle noise is multiplicative noise. In order to reduce the speckles in ultrasound images effectively, we develop speckle reducing bilateral filter.

### Speckle reducing bilateral filter

Generally speaking, noise can be modelled by an additive model or a multiplicative model. Additive noise model is the simpler case of the two and can be described by a linear model

*J*(*X*) = *I*(*X*) + *n*(*X*)     (3)

where *J*(·) is the noised image, *I*(·) is the original image and *n*(·) is the noise. Multiplicative noise is generally expressed by a multiplicative model

*J* (*X*) = *I* (*X*) * *n*(*X*)     (4)

It is well known that multiplicative noise appears much worse in bright image regions than dark regions since it multiplies the gray intensities.

Speckle noise is generally treated as multiplicative noise and can be modelled using equation (4). Thus, compared with other types of noise, speckle noise is generally difficult to be removed. Our research below shows that the conventional bilateral filter described in equation (1) and (2) generally gets bad results when it is applied to speckle reduction directly. Thus, the bilateral filter described in (1) and (2) needs improvement or enhancement so that it can be applied to reduce the speckles in images effectively. In order to do this, we will first analyze the behavior of  of the bilateral filter in equation (1) in a homogenous region for both additive noise and multiplicative noise, then we will propose an adaptive bilateral filter for speckle reduction.

Let *J*(*Y*) and *J*(*X*) be two different pixels from image *J.* If *J* is corrupted by additive noise, then we can use equation (3) to compute the difference between these two pixels

||*J*(*Y*) - *J*(*X)*|| *= ||I*(*Y*) + *n(Y*) -*I*(*X*) - *n*(*X*)||     (5)

If both *J*(*Y*) and *J*(*X*) are from the same homogenous region, then we have *I*(*Y*) = *I*(*X*), thus

||*J*(*Y*) - *J*(*X)|| = ||n*(*Y*) - *n*(*X*)||     (6)

Equation (6) means that the difference between any two pixels from the same homogenous region is only related to the difference of the noise. If *J* is corrupted by multiplicative noise, then we can use equation (4) to compute the difference between these two pixels. From equation (4), we have

||*J*(*Y*) - *J*(*X)|| =* ||*I*(*Y*) * *n*(*Y*) - *I(X*) * *n*(*X*)||     (7)

Similarly, if both *J*(*Y*) and *J*(*X*) are from the same homogenous region, then we have *I*(*Y*) = *I*(*X*), thus

||*J*(*Y*) - *J* (*X*)|| = ||*I*(*X*)||||*n*(*Y*) - *n*(*X*)||     (8)

From equation (8), we can understand that the difference between two pixels in the same homogenous region(in the image corrupted by multiplicative noise) is not only related to the difference of the noise. It also depends on the intensity of the region. As is seen in equation (8), the difference is big when the intensity of the region is big while the difference is small when the intensity of the image is small.

The above analysis shows the bilateral filter described in (1) and (2) is not suitable for removing speckle noise, which is multiplicative noise. The reason lies in the difference of the corrupted image has different distributions in different homogenous regions. For example, if *σ _r_*^2^ is fixed in the processing, when *σ _r_*^2^ is set to be big, the edge in lower intensity regions will be removed, while the noise can’t be removed in the higher-intensity regions when *σ _r_*^2^ is set to be small. Thus, in order to develop an effective bilateral filter to remove the speckle, we need to develop a new representation of the difference. Dividing each side of equation (8) by ||*J* (*X*)||, in the homogenous regions, we have

     (9)

Equation (9) shows that the normalized difference is only related to the noise and doesn’t depend on the intensities of the region. Thus, the proposed adaptive bilateral filter can be expressed as follows

     (10)

where

     (11)

Bilateral filter is famous because it is non-iterative, however, the non-iterative bilateral filter doesn’t yield good results. In order to improve its effectiveness, we use iterative bilateral filter. The basic idea of iterative bilateral filter is to use the filtered image obtained by equation (10) as the input of equation (1) and implement it many times, the mathematical expression is as follows:

     (12)

where

     (13)

Where . Experiments show that iterative bilateral filter gives much better results than the non-iterative bilateral filter.

### Cattle follicle segmentation

In order to analyze and monitor the reproduction of cattle, the acquisition of some quantitative parameters is very important. These parameters include diameters, areas and perimeters of the follicles. These parameters can be used to monitor the development and maturity of follicles. In order to get these parameters, we need to segment the follicles.

Many image segmentation methods have been proposed, which includes histogram based methods, edge detection based methods, region based methods, active contour model based methods, etc. Active contour model based methods have drawn a lot of attention in the past decade because of their significant advantages. In this paper, we adopt active contour model based method for the segmentation of the follicles. An active contour or a snake [19] is defined as a controlled continuity contour that is attracted to salient image features. However, there are some disadvantages related to the original model. Thus, many improved active models have been proposed based on the original model. The gradient vector flow (GVF) model is one of them [20][21]. GVF model is designed to overcome one of the disadvantages of original model, i.e. the original model is sensitive to the initialization of the snake. In GVF model, GVF fields are computed by another diffusion process, which can be implemented by minimizing the following energy function [20][21]:

     (14)

where *g* is a decreasing function of the edge-force magnitude and is defined as follows:

     (15)

Here *k* is a non-negative smoothing parameter for the field (*u*, *v*)*.* The functional described by equation (15) smoothes the force field (*u*, *v*) only when the edge strength is low. Solving the energy functional optimization problem in (14), we can obtain the generalized gradient vector flow, which can be used as external forces that attract the snake to the follicle boundary [20][21].

GVF provides external forces for a snake model, we also need internal forces to smooth the contour. In this paper, we use B-spline to represent the contour instead of the real internal forces. B-spline has been used in snake model in several applications and get pretty good results [22][23][24]. Let the control points be denoted by *P*_0_ through *P_m_*. The knot-value sequence is a non-decreasing sequence of knot values *t*_0_ through *t_m_*_+4_, and *Q_i_* is a curve segment defined by control points *P_i-_*_3_, *P_i-_*_2_, *P_i-_*_1_, *P_i_* and blending functions *B_i-_*_3,4_, *B_i_*_-2,4_, *B_i_*_-1, 4_, *B_i_*_, 4_ (*t*) as follows [22]:

*Q_i_* (*t*) = *P_i-_*_3_ · *B*_i-3, 4_ + *P*_i-2_ · *B_i_*_-2,4_ + *P_i_*_-1_ · *B_i_*_-1,4_ + *P_i_* · *B_i_*_,4_ (*t*)	(16)

where 3 *≤ i ≤ m* and *t_i_ ≤ t ≤ t_i+_*_1_. The blending functions can be obtained using recursion as follows [22]:

     (17)

     (18)

When *p*=4, we obtained the blending function of cubic splines. The GVF snake with B-spline is called B-spline GVF snake [23][24][25].

For the segmentation of the follicles, we initialize the B-spline GVF snake using a circle inside each follicle. The circle is represented by B-spline and the number of control points is set to 48 in this paper. Then, starting from the initial contour, the GVF is used to drive the contour to the boundary of the follicle. The evolution of the contours is the same as that in the B-spline GVF snake in single scale proposed by [24].

## Results

### Results from Synthetic Images

**Figure 1:**
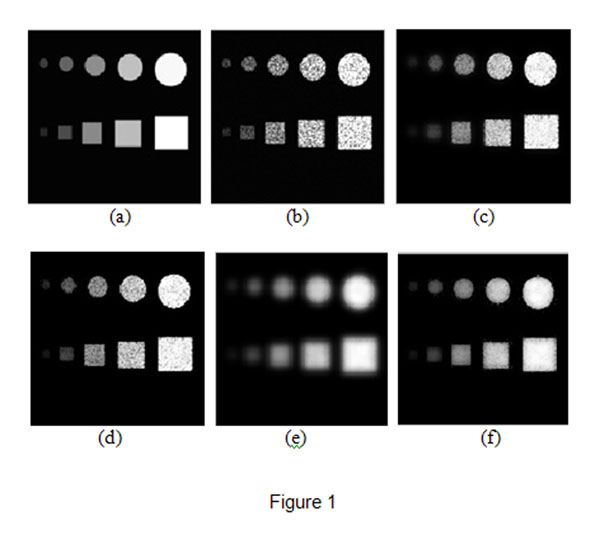
**Synthetic image and despeckling results.** (a) original synthetic image; (b) multiplicative noise image; (c) the best result by the conventional bilateral filter(*σ_r_* = 3 *σ_d_* =0.3); (d) the result by the proposed bilateral filter(*σ_r_*= 3 *σ_d_* =0.3); (e)the result by the conventional bilateral filter(*σ _r_* = 3 *σ _d_* =0.7); (f) the best result by the proposed bilateral filter(*σ_r_* = 3 *σ_d_* =0.7).

To test the effectiveness of the proposed bilateral filter, we used both conventional bilateral filter and the proposed bilateral filter to process the synthetic image with speckles and compare the results. Fig.1(a) is the original image and Fig. 1 (b) is the corrupted image by speckles with mean 0 and variance 0.075. In order to demonstrate the effectiveness of the proposed filter and evaluate its performance in speckle reduction and edge preservation, we employed three measures in the experiments for comparison. These three measures are: normalized mean square error (NMSE), noise suppression measure *α* and edge preservation parameter *β*[26]. The NMSE is defined as [26]

     (19)

where I_0_ and I are the original image and the corrupted image, respectively, N is the pixel number of the image I_0_ (or) I,  and  are the means of I and I_0_, respectively. The NMSE generally represents the difference between the original image and the processed image. The noise reduction measure is defined as [26]

     (20)

where

     (21)

The edge preservation parameter is given by [26]

     (22)

where Δ is the Laplacian operator. Higher *α* and *β* represent better performance in noise reduction and edge preservation.

**Figure 2:**
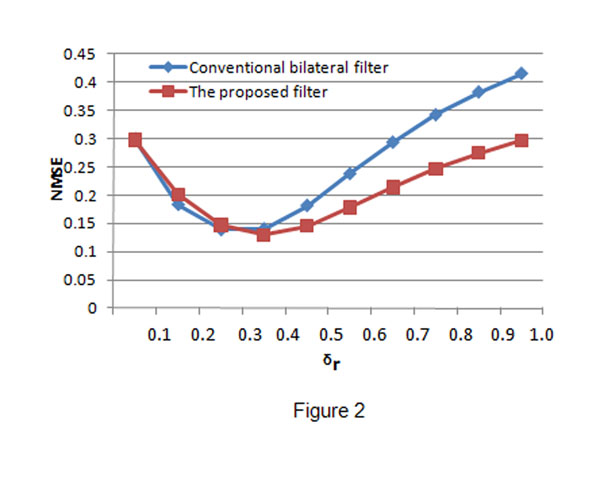
**NMSE value comparison.** The blue line shows the NMSE values obtained by the conventional bilateral filter and the red line shows the NMSE values obtained by the proposed bilateral filter. Both filters have big NMSE values when *σ _r_* is small. The proposed filter has smaller NMSE values than the conventional bilateral filter after *σ_r_* reaches the optimal point.

**Figure 3:**
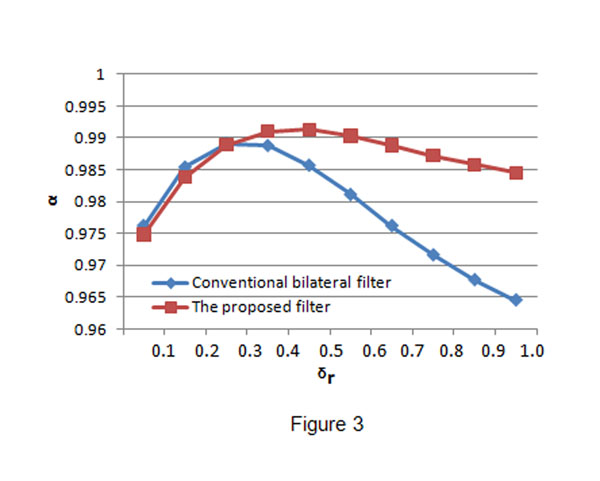
**Noise reduction measurement comparison.** The blue line shows the *α* values obtained by the conventional bilateral filter and the red line shows the *α* values obtained by the proposed bilateral filter. The proposed filter has much better performance in noise reduction than the conventional bilateral filter after *σ_r_* reaches the optimal point.

**Figure 4:**
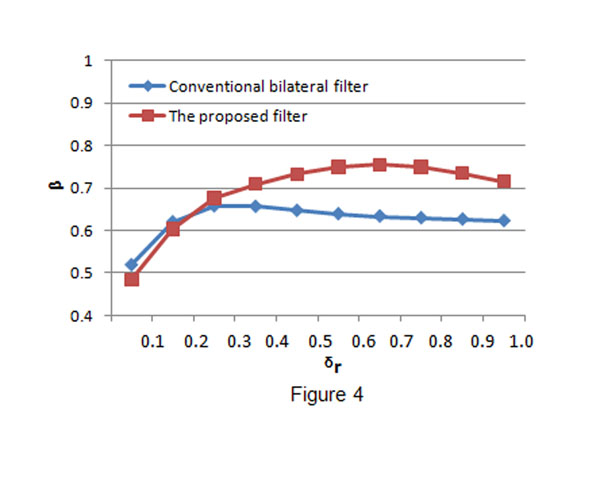
**Edge preservation measurement comparison.** The blue line shows the *β* values obtained by the conventional bilateral filter and the red line shows the *β* values obtained by the proposed bilateral filter. The proposed filter has much better performance in edge preservation than the conventional bilateral filter after *σ_r_* reaches the optimal point.

The conventional bilateral filter and the proposed bilateral filter were applied to process the speckled images. In both filters, *σ _d_* was fixed to be 3 and *σ _r_* was set to be ranged from 0.1 to 1.0. We use the iterative scheme in the conventional bilateral filter and the proposed filter, iteration is 5 for the two filters. The values of NMSE, *α* and *β* obtained from the processed images are given in Fig.2, Fig.3 and Fig.4, respectively. From the figures, we can find that when *σ _r_* is small, we have big NMSE values, small *α* and *β* values for both filters. This result means that both filters have poor performance in noise suppression and edge preservation when *σ _r_* is small. When *σ _r_* increases, the performance (in both noise reduction and edge preservation) of both filters will be improved and then the best performance is achieved when some *σ _r_* is reached. We call the *σ _r_* which makes a filter have the best performance as the optimal point, denoted by *σ _r_^T^*. Obviously, the two filters have different optimal points and the performance of a filter will become worse when *σ _r_* is bigger than its optimal point *σ _r_^T^*. The above quantitative measurement also reveals that the conventional bilateral filter behaves better than the proposed bilateral filter when *σ _r_* is small, and the proposed bilateral filter outperforms the conventional bilateral filter quickly with the increase of *σ _r_*. However, at the optimal points, the proposed bilateral filter has better performance than the conventional bilateral filter.

Fig.1(c) and 1(f) are the best results obtained by the conventional bilateral filter with *σ _r_* = 0.3 and the proposed bilateral filter with *σ _r_* = 0.7 respectively. In Fig. 1(c), there are still many speckles while the smaller objects are blurred and nearly smeared out. However, in Fig. 1 (f), most of the speckles are removed and the objects are preserved. We also compared the results obtained by the two filters with the same parameters. Fig. 1 (d) is the result obtained by the proposed filter with *σ _r_* = 0.3, which is the same as the setting in Fig.1(c). The NMSE, *α* and *β* are 0.1391, 0.9891 and 0.6571 in Fig.1(c), while the measurements are 0.1474, 0.9889, 0.6769 in Fig.1 (d). It shows that there are more speckles in Fig.1 (d), but the smaller objects and edges are clearer than that in Fig. 1(c). Fig.1 (e) is the result obtained by the conventional bilateral filter with the same *σ _r_* = 0.7 as the result in Fig. 1(f). It illustrates that speckles are removed effectively and all edges are retained, however, all objects are blurred heavily in Fig.1(e), especially the smallest circle and rectangle are smeared out. The measurements, NMSE, *α* and *β* are 0.2937, 0.9762 and 0.6335 in Fig.1 (e), while the three measurements are 0.2146, 0.9888, 0.7547 in Fig.1(f).

All of the above experiments show that the proposed bilateral filter can achieve better performance in noise removal and edge preservation than the conventional bilateral filter.

### Results from real ultrasound Images

**Figure 5:**
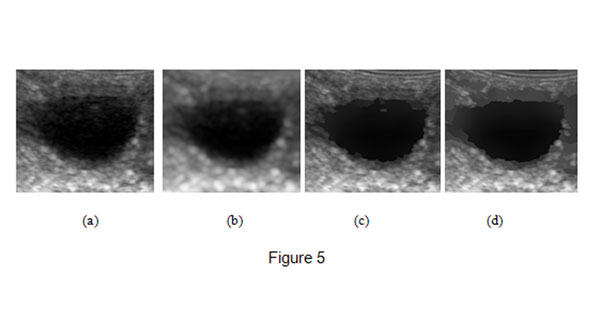
**Denoised ultrasound images of cattle follicles.** (a) shows the origianl image and (b),(c),(d) show the results obtained by Gaussian filter(standard deviation of the Gaussian kernel is 3.0 and window size is 9), the conventional bilateral filter and the proposed filter respectively(*σ_r_* = 3 *σ_d_* =0.5,iteration=40).

In this subsection, we will compare the proposed bilateral filter with Gaussian filter and the conventional bilateral filter in speckle reduction using real ultrasound images. Fig.5 shows the original image and the results obtained by the three filters. Although Gaussian filter may reduce the speckles in the images as seen in Fig.5 (b), the edges and details are very blurred. The useful details in the processed image (see Fig. 5(c)) obtained by the conventional bilateral filter are retained, but there are still many speckles. In Fig.5 (d), we know that the proposed filter can reduce speckles effectively while preserve useful edges and details.

To compare and evaluate the three filters quantitatively, we used them to reduce the speckles in real ultrasound image and then calculated the contrast of the homogenous region and edges in the image. A good filter should preserve the edges and reduce speckles in the image, which means the contrast in homogenous region should be low while the contrast in edges should be high. The contrast measure used in this paper is the measure adopted in [27], which is defined as

     (16)

where *c*(*x, y*)*,* the local contrast at pixel (*x, y*), is the Laplacian operation

*c*(*x*, *y*) = 4*I*(*x*, *y*) - {*I*(*x* - 1, *y*) + *I*(*x*, *y -* 1) + *I*(*x* + 1, *y*) + *I*(*x*, *y +* 1)} (17)

where *I*(*x, y*) is the pixel intensity value at pixel (*x*, *y*) of an image, *w* is a region or a set of edge points, and *m* is the number of the pixels in the region or edge points.

**Figure 6:**
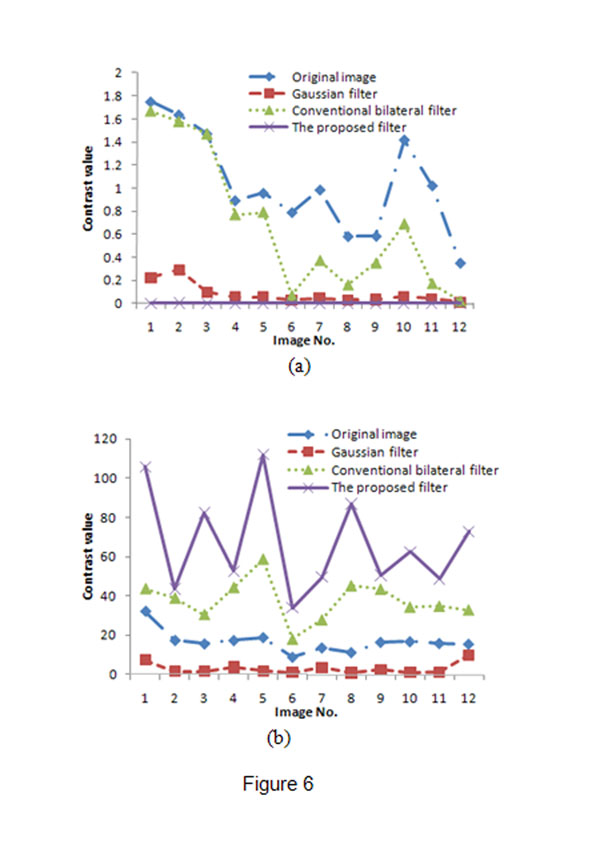
**Contrast comparison.** (a) Contrast of homogenous region; (b) Contrast of edge points set.

Fig.6 illustrates the contrast values in the preselected homogenous regions and the preselected sets of edge points of 12 follicle images. For the homogenous regions, Fig. 6 (a) shows that the contrast values of the regions obtained by Gaussian filter are smaller than those obtained by the conventional bilateral filter or the proposed bilateral filter. Besides, the proposed bilateral filter obtained the smallest contrast values (all are less than 0.04). These results show that the proposed bilateral filter can achieve the best performance in speckle reduction in homogenous regions. For the set of edge points, Fig.6 (b) shows that Gaussian filter has the smallest contrast values, which indicates that most of the edges have been smeared out. Although the conventional bilateral filter has higher contrast values in the set of edge points, the proposed filter has the biggest contrast values, which means it has higher performance in edge preservation.

**Figure 7:**
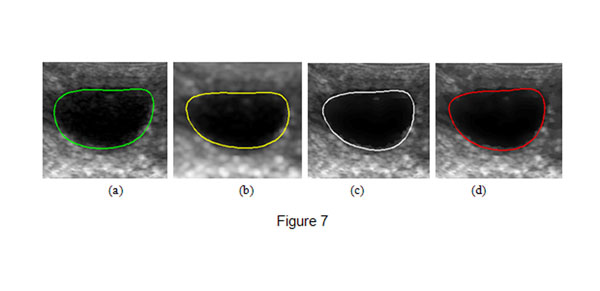
**Final boundaries of follicles.** (a) shows the final contours of the follicle obtained from the origianl image and (b),(c),(d) show the contours of the follicle obtained from the images filtered by Gaussian filter,the contional bilateral filter and the proposed filter respectively.

After the images were processed, we applied B-spline snake [28] to extract the boundaries of the follicles. Fig.7 shows the experimental results. Fig.7 (a),(b),(c),and (d) show the boundaries of the follicles extracted by B-spline snake from the original images, the images processed by Gaussian filter, the contional bilateral filter and the proposed filter, respectively. Fig.7(a) shows that the final contour is away from the boundary of the follicle due to the speckles. Although there are less speckles in Fig.7(b), the final contour is also away from the real boundary because the edges are blurred by Gaussian filter. The result of Fig.7(c) is very close to the real boundary of the follicle than the contour in Fig.7(a) and Fig.7(b), but it is still affected by speckles. Fig.7 (d) shows that B-spline snake can accurately locate the real boundary of the follicle filtered by the proposed algorithm.

In order to evaluate the segmentation results, we adopted the segmentation metric, Pratt's quality measurement metric (FOM), which is defined as [29]

     (18)

where I_A_ is the number of boundary pixels delineated by an automatic segmentation method, I_I_ is the number of boundary pixels delineated by the technicians. *d(i)* is the Euclidean distance between a boundary pixel of ground truth or delineated by the technicians and the nearest boundary pixel extracted by automatic segmentation, and γ is a scaling constant(0.05 in our experiments).

**Figure 8:**
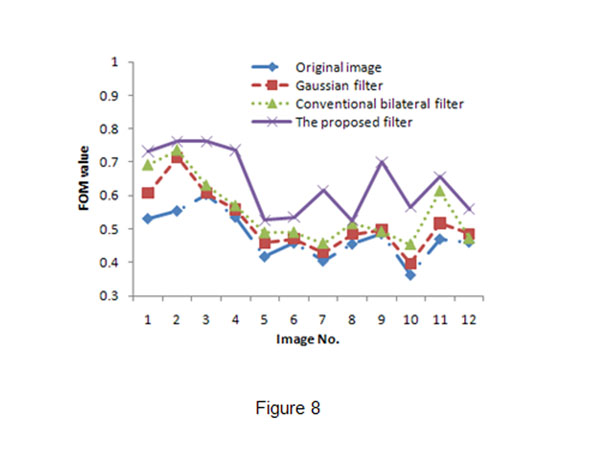
**Comparison of FOM values.** The blue, red, and purple lines show the FOM values for the follicle segmentation using the original image, the filtered image by Gaussian filter, the filtered image by conventional bilateral filter, and the filtered image by the proposed filter(with the best FOM value).

Fig.8 shows the FOM values of the 12 images processed by different filters. We can see that the Gaussian filter could improve segmentation, the conventional bilateral filter and the proposed filter achieved better FOM values than Gaussian filter. However, the proposed bilateral filter outperformed the other two filters.

## Discussion

Bilateral filter is a powerful technique in image de-noising due to its stability, and simplicity. The basic idea of bilateral filter is to replace a pixel value by a weighted average of its neighbours in both space and range (pixel values). However, the conventional bilateral filter performs poorly on ultrasound images due to the speckles. From the multiplicative noise model, we investigated a normalized scheme based on the conventional bilateral filter so as to remove the speckles effectively while preserving useful details. For bilateral filter, the parameters including *σ _d_*^2^ and *σ _r_*^2^** play a vital role in noise removal and edge preservation. It has been demonstrated that the optimal *σ _d_*^2^ is relatively insensitive to noise variance while the optimal *σ _r_*^2^ value changes significantly as the noise standard deviation changes. To investigate the performance of bilateral filter with different values of *σ _r_*^2^, we applied the bilateral filters on synthetic images and used three quantitative measures including NMSE, noise reduction measure and edge preservation measure for analysis and comparison. We can see that the proposed method is more robust and effective than the conventional bilateral filter. The above three measures can be used for parameter selection of bilateral filters. However, since the ideal signals or non-noised images are usually unknown for real biomedical images, we should define other measures such as local contrast of homogenous regions and edge points set. Our local contrast is more robust and effective for algorithm evaluation in noise reduction and details preservation. This kind of measure can be adopted for parameter selection in bilateral filters when the filters are applied to real images. We compared the proposed filter with the conventional bilateral filter and Gaussian filter. Although Gaussian filter can reduce noises more or less, most of the edges and details have been smeared out. The conventional bilateral filter behaved poorly in speckle reduction. Experimental results of real ultrasound images of follicles illustrate that our proposed algorithm could obtain the best performance.

## Conclusions

We presented a normalized bilateral filter for speckle reduction in ultrasound images for follicles segmentation. We compared the conventional bilateral filter with the proposed filter using synthetic speckled images and demonstrated its good performance in speckle reduction and edge preservation. Besides, we also tested the proposed filter, the conventional bilateral filter and Gaussian filter using real ultrasound images of cattle follicles. The contrast values of homogenous regions and edge points set demonstrated the proposed algorithm could achieve the best performance. The segmentation experiments also proved that B-spline snake can accurately find the boundary of the follicles from the filtered images by the proposed method. Experimental results validated the effectiveness and the accuracy of the proposed filter in noise reduction and edge preservation for follicle segmentation.

## Competing interests

The authors declare that they have no competing interests.

## Authors' contributions

JT developed the algorithm and wrote non-results part of the paper. SG implemented the algorithm and wrote the result part. QS attended to develop the algorithm. YD and DZ helped data analysis. All authors read and approved the final manuscript.
